# Time- and distance-resolved robotic imaging of fluid flow in vertical microfluidic strips: a new technique for quantitative, multiparameter measurement of global haemostasis†

**DOI:** 10.1039/d3sd00162h

**Published:** 2023-10-17

**Authors:** Rüya Meltem Sarıyer, Kirandeep Gill, Sarah H. Needs, Daniel Hodge, Nuno M. Reis, Chris I. Jones, Alexander D. Edwards

**Affiliations:** a Reading School of Pharmacy, University of Reading Whiteknights Reading RG6 6UB UK r.sariyer@pgr.reading.ac.uk kkgill@uvic.ca s.h.needs@reading.ac.uk a.d.edwards@reading.ac.uk +44 (0)118 378 4253; b Department of Chemical Engineering and Centre for Biosensors, Bioelectronics and Biodevices (CBio), University of Bath Bath BA2 7AY UK n.m.reis@bath.ac.uk; c Reading School of Biological Sciences, University of Reading Whiteknights Reading UK d.j.hodge@pgr.reading.ac.uk c.i.jones@reading.ac.uk; d School of Electronics and Computer Science, University of Southampton Highfield Southampton SO17 1BJ UK

## Abstract

Measuring the complex processes of blood coagulation, haemostasis and thrombosis that are central to cardiovascular health and disease typically requires a choice between high-resolution low-throughput laboratory assays, or simpler less quantitative tests. We propose combining mass-produced microfluidic devices with open-source robotic instrumentation to enable rapid development of affordable and portable, yet high-throughput and performance haematological testing. A time- and distance-resolved fluid flow analysis by Raspberry Pi imaging integrated with controlled sample addition and illumination, enabled simultaneous tracking of capillary rise in 120 individual capillaries (∼160, 200 or 270 μm internal diameter), in 12 parallel disposable devices. We found time-resolved tracking of capillary rise in each individual microcapillary provides quantitative information about fluid properties and most importantly enables quantitation of dynamic changes in these properties following stimulation. Fluid properties were derived from flow kinetics using a pressure balance model validated with glycerol–water mixtures and blood components. Time-resolved imaging revealed fluid properties that were harder to determine from a single endpoint image or equilibrium analysis alone. Surprisingly, instantaneous superficial fluid velocity during capillary rise was found to be largely independent of capillary diameter at initial time points. We tested if blood function could be measured dynamically by stimulating blood with thrombin to trigger activation of global haemostasis. Thrombin stimulation slowed vertical fluid velocity consistent with a dynamic increase in viscosity. The dynamics were concentration-dependent, with highest doses reducing flow velocity faster (within 10 s) than lower doses (10–30 s). This open-source imaging instrumentation expands the capability of affordable microfluidic devices for haematological testing, towards high-throughput multi-parameter blood analysis needed to understand and improve cardiovascular health.

## Abstract 2

High throughput, automated, time-lapse imaging for multiparameter haemostasis.

## Graphical abstract

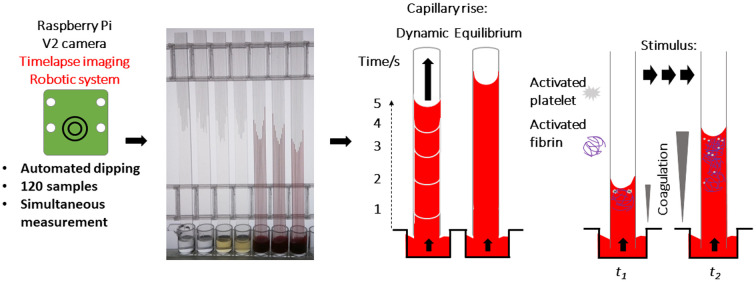

## 1. Introduction

Haemostasis and thrombosis play a major role in cardiovascular health and disease. Measurement of these processes remains critical to health and wellbeing; both for optimising cardiovascular disease treatments in acute and chronic clinical settings, and in research progressing our understanding the cardiovascular system. Some assays measuring coagulation, such as prothrombin time, or platelet function, for example, aggregometry,^1^ have been automated, but most analysis still relies on complex laboratory assays which can be time-consuming, expensive, laborious and require personnel with specific technical training.^1–10^ There is therefore a need for low-cost disposable devices that enable platelet function testing at the point-of-care or point-of-need.

Recent deeper multiparameter analysis has been established in small studies using current laboratory tools such as time-resolved cytometry or multiparameter phenotyping of platelets, demonstrating that quantitative concentration–response measurement provides significant novel insight,^11,12^ indicating the need for greater depth of analysis, ideally in scalable measurement systems. The difficulties of analysing haemostasis and thrombosis at scale are exacerbated by the need to perform tests shortly after phlebotomy, so the blood donor needs to visit a laboratory research site. Although some lab tests can be conducted on stored samples or from biobanks (*e.g.* serology, genetics), it is not possible to store live platelets in blood samples and transport them to laboratories without dramatically altering their phenotype. Such measurements must typically be completed within 1 hour of drawing blood, even when using automated analysers,^13^ making larger-scale use challenging. As a result, clinical use remains limited, and large-scale research studies are restricted to the few narrow parameters (*e.g.*, platelet count, prothrombin time) that can be measured easily. This impedes our ability to perform essential population studies that combine detailed platelet and coagulation measurements with other large-scale techniques including genomics, proteomics, metabolomics and data science. Alternative techniques are therefore needed to answer questions such as the underlying causes of altered coagulation pathways or platelet function by age, diet, or during infection that have not yet been fully understood.^14–18^ The ideal tools would measure multiple coagulation and/or platelet parameters in response to panels of stimuli, use inexpensive consumables and avoid costly laboratory instrumentation, and must be easy to use without training whilst capturing as much data as possible.

To this end, a range of microfluidic devices have been developed to measure many elements of blood haemostasis, including soluble and cellular factors (*e.g.* fibrinogen and platelets). For example, fibrinogen has been measured in whole blood using microfluidic channels.^19^ Migration-based platelet aggregation in microdevices allowed measurement of platelet activation.^20–23^ A two-channel microfluidic device with a collagen-coated section examines the effects of stirring on shear-induced platelet activation by measuring migration distance.^20,21^ Another two-channel microfluidic chip tests platelet aggregation induced by a combination of two stimuli, adenosine diphosphate (ADP) and collagen, measured the migration distance by adding stimulated blood to the microchip.^22^ Paper-based microfluidic lateral flow devices have been used to monitor blood coagulation, with blood coagulation initiated by adding CaCl_2_ solutions, and following migration time of blood. A linear relationship between the migration time and the viscosity of the fluid was observed.^23^ Larger numbers of channels allow multiplexing measurement of more parameters, for example use of transparent melt-extruded 10 channel devices instead of porous media allows different changes to be detected, for example red cell aggregation for ABO blood typing that is visible from digital scans alongside delayed capillary rise.^24^ These studies illustrate the general suitability of microfluidic systems for measuring blood function; but for microfluidic assays to fulfil their potential, larger scale application in research and diagnosis is now needed.

Blood rheology is complex, with multiple studies showing how the viscosity of blood varies with shear rate^25^ whereas plasma (without cells) shows rheological behaviour closer to a Newtonian fluid with viscosity independent of shear rate.^26^ Hemodynamics affect platelet function and coagulation, with wall shear rate and wall shear stress being the major parameters characterising laminar flow of blood *in vivo.*^27^ For humans, typical average wall shear rates range from 40 to 500 s^−1^ in blood vessels experiencing pulsative flow, and arterial thrombosis formation is observed in human arteries at wall shear rates as low as 100–200 s^−1^.^27^ Flow chambers have therefore been widely used for studying and reproducing the process of thrombosis formation *in vitro*,^28^ with channel size plus flow rate affecting shear and dynamic viscosity during blood flow; channel sizes of 100–300 μm have been recommended.^28^ Smaller sizes result in dominance of platelet-wall interaction over platelet–platelet interaction. A transient viscosity study showed that blood viscosity changes quickly during coagulation at different shear rates (20–80 s), with the time-to-gel point in ranging from 60 to 120 s; the gel point is defined as the time at which at least a 20% increase in blood viscosity is observed.^29^ The same authors showed blood viscosity plateauing at shear rate of 80 s^−1^. Several studies have proposed microcapillary rheometers that measure rheology with very small sample volumes over wide of shear rate ranges (10–10^5^ s^−1^).^30–32^ Although very precise, these require complex instrumentation.

To gain maximal data from microfluidic devices, portable and inexpensive instrumentation is needed that captures time and position-resolved data. Ideally imaging should be coupled to control of sample processing, simplifying use and ensuring consistency of captured data. Digital imaging has never been smaller, cheaper, or higher performance, with increasingly sophisticated mass-manufactured CMOS digital cameras capable of quantifying a wide range of assay readouts. These can be combined with free open-source hardware and software (FOSH and FOSS), to develop affordable scientific instruments that replace expensive bespoke laboratory equipment.^33,34^ The growth of FOSH is following a previous rise of FOSS with a 20 year lag^35^ with potential to reduce experimental costs for science and engineering.^34^ Open source systems make scientific equipment more accessible not only for diverse research but for a wider range of users,^36^ for example the Raspberry Pi computer and camera has been used to develop low-cost open-source multi-fluorescence imaging^33^ and controlled temperature and pressure monitoring systems.^37^ Alongside imaging, the Raspberry Pi has flexible outputs that allow easy and inexpensive addition of robotic sample manipulation and light control.^38^ Affordable 3D printing allows cheap and rapid replication and iterative prototyping of complex instruments.^39^ 3D printed devices can simplify sample preparation and nucleic acid amplification.^40^ 3D printed structures combined with CMOS digital cameras are ideal for microscopy, illustrated by the openflexure system^41^ and digital holographic microscope.^42^ Open-source hardware can ensure technological advances in digital imaging drive rapid scientific progress.^38^

Here, we demonstrate for the first time that integration of multiplexed ‘dip stick’ microfluidics^24^ with a time and distance-resolved robotic imaging technique, allows detailed analysis of fluidic properties of blood, and most importantly capturing any changes of those properties during and immediately following stimulation with agents that trigger activation of blood (*e.g.* coagulation). To allow low cost open-source robotic digital imaging instrumentation to facilitate large-scale screening, we integrated it with mass-produced melt-extruded microfluidics to couple capture of high-density data with an ability to multiplex stimuli. The vertical “dip-stick” format simplifies automation of sample testing because it requires no liquid handling, in contrast to horizontal devices where a liquid sample must be added into a well; with vertical dipping, devices can simply be lowered into the sample using a robotic arm or a servo motor. Furthermore, many devices can be dipped simultaneously with a single servo. To assess this combination, we tested activation of fibrin polymerisation with thrombin. By imaging instantaneous capillary rise and equilibrium capillary height in vertical array of hydrophilic microcapillaries, it was possible to extract quantitative information about blood coagulation in whole blood samples by best-fitting high-density experimental data to pressure balance models of transient and equilibrium fluid flow in vertical hydrophilic microcapillaries. With the initial stage of capillary rise showing mean superficial flow velocities of 10–30 mm s^−1^, corresponding to theoretical wall shear rates of 100–600 s^−1^, blood viscosity can be assumed independent of shear rate. Pressure balance models were validated using known glycerol–water mixtures, and we show the technique is able to quantify differences in viscosity and surface properties (surface tension and contact angle) between water, plasma and whole blood. This study demonstrates the power of inexpensive (<£300) open source instrumentation to expand data captured from microdevices, opening the door to larger scale measurement of the dynamics of haemostasis.

## 2. Experimental section

### 2.1. Materials and test strip production

HEPES buffered saline was made using Fisher BioReagents components (Leicestershire UK); thrombin from bovine plasma (product T4648), Tween 20 (product P1379, Sigma Aldrich, UK), glycerol (Sigma Aldrich, UK), and polyvinyl alcohol (MW 146 000–186 000, >99% hydrolysed), were from Sigma Aldrich (Dorset, UK); ultrapure water was from an Elga Veolia Purelab Chorus (Elga LabWater, UK). Microcapillary film made from fluorinated ethylene propylene of 160, 200 and 270 μm diameter was obtained from Lamina Dielectric Ltd (Billingshurst, UK). Fluoropolymer microcapillary film (MCF) was coated internally by incubation with 10 mg mL^−1^ high molecular weight polyvinyl alcohol as previously described.^43,44^ This transforms the inner hydrophobic surface of FEP microcapillaries to hydrophilic and allows aqueous sample uptake by capillary action by simply dipping the ends of the ‘dip stick’ strips into the sample. Hydrophilic coated MCFs of 3 different mean inner diameters (160, 200 and 270 μm), all cut to 60 to 100 mm lengths with a sharp blade, were used to perform kinetic experiments of glycerol–water mixtures with well-defined fluid properties, and of the indicated blood components. Since the capillaries were elliptical,^24^ the major and minor axis of individual capillaries was determined from digital micrographs of the ends of strips cut with a sharp blade to reduce distortion, and analysed with ImageJ (NIH, USA^45^).

### 2.2. High-throughput microfluidic blood analysis system using open source robotic imaging

**Fig. 1:**
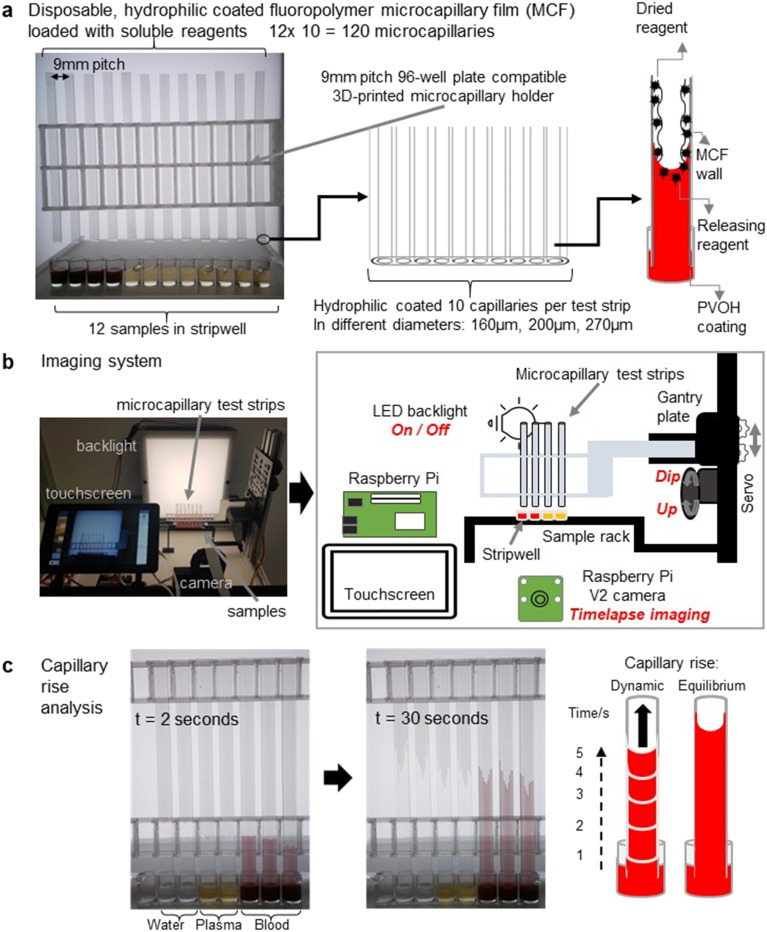
High throughput time-resolved analysis of capillary rise in an array of mass-produced ‘dip stick’ microfluidic devices using open-source Raspberry Pi robotic imaging system. (a) Disposable, reagent-loaded MCF test strips analyse 12 samples in 96 well-plates using 3D printed holders. Hydrophilic coated MCF has 10 capillaries per strip, allowing up to 10 replicas per sample. Individual capillaries can be loaded with different reagents allowing up to 120 different tests to be performed simultaneously. Dried reagents within the capillaries are released and mixed upon dipping of the strip into a liquid sample such as blood as the liquid sample rises upwards. (b) The flexible and customisable robotic microfluidic system (RMS) combines digital camera with accurate sample movement and controlled lighting. Light, sample dipping and camera are operated by a Python script. Strips were mounted on a gantry plate for vertical movement, and dipped into the samples *via* servo motor. (c) Throughout the test, the Raspberry Pi camera takes time-lapse images of 120 individual capillaries (12 strips each of 10 microcapillaries). Kinetic data of capillary flow dynamics, especially instantaneous velocity, are obtained by image analysis.

The open-source, high-throughput microfluidic blood analysis system (Fig. 1b) has imaging, lighting, and sample dipping controlled by simple Python scripts. It combines dip-and-test microfluidic devices with a low-cost (approximately £300), open-source, customizable robotic microfluidic blood analysis platform named “Robotic Microfluidic System” (RMS). With an overall dimension of 350 mm × 415 mm × 288 mm, it was developed to measure the fluid properties of blood during coagulation in response to panels of stimuli, by time-resolved digital imaging of vertical capillary rise. V-slot aluminium extrusions of the required lengths (Ooznest, UK) were used to build the system, connected with off-the-shelf open source OpenBuilds hardware. Test strips were mounted on a gantry (Ooznest, UK) that moved vertically on V-slot extrusion to provide linear motion of dipping of the ‘dip stick’ microfluidic strips. The system combines a Raspberry Pi model 4 (4Gb RAM model), Raspberry Pi Camera V2.1 and Raspberry Pi 7′′ touchscreen (Raspberry Pi (Trading) Limited, Cambridge, UK), with a LED lightbox made from 12v LED strips (RS components, UK), and holders for MCF test strip moved vertically by a standard miniature servo motor (Parallax Inc, California, USA) with a 3D printed cam screwed onto the servo horn, to dip into 96-well plate or strip-wells. The lighting and camera conditions in the system was designed to provide the optimum image resolution (Fig. S1†). Holders for the servo, batteries, relay, test strip and well-plate were 3D printed in polylactic acid (PLA) on a Prusa i3 MK3 (Prusa Research, Prague, Czech) using default printer settings. Opal acrylic light diffuser sheet for the front, black cast acrylic sheet for the back and V-slot aluminium linear rail extrusions for the frame were used in the build of the lightbox (Fig. 1b). A Grove single relay unit was used for the Raspberry Pi GPIO to control light source switching. Technical specifications, user manual, 3D design files, full material list, build instructions, and software codes for the RMS are available in GitLab^46^ and published in Zenodo (DOI: https://doi.org/10.5281/zenodo.6617301).

### 2.3. Preparation of blood samples

Blood samples were collected from healthy donors using citrate blood collection tubes (BD Vacutainer®) with 3.2% buffered sodium citrate solution, which reversibly prevents coagulation; after collection samples were stored at 37 °C for a maximum of 3 hours before testing. A minimum of 20 μL was required per 60–100 mm MCF strip although 200 μL was added to each well to ensure complete submersion of the test strip end. Prior to testing blood was incubated at 37 °C in a water bath for a minimum of 5 minutes. Where required, the citrated blood was centrifuged at 102 × *g* for 20 min at 20 °C to separate platelet-rich plasma (PRP) from the whole blood (WB). PRP was subsequently centrifuged at 1413 × *g* for 10 min at 20 °C to obtain platelet-poor plasma (PPP). Separately, WB was also centrifuged at 500 × *g* for 10 min at 4 °C and red cell pellet washed 3 times with HEPES buffered saline (HBS) to isolate red blood cells (RBC) that were resuspended in HBS.

### 2.4. Kinetic analysis of glycerol–water mixtures and blood components

Mixtures of glycerol with ultrapure water were prepared at 0, 20, 40 and 60% w/w and were placed under the hydrophilic MCF test strips in a clear flat-bottomed 96-well microplate. Sets of MCF strips were fitted on a 3D printed holder (Fig. 1a) and clipped into the gantry system. Samples (pure water, HBS, PPP, PRP, RBC and WB) were likewise tested with MCF test strips. In all cases a python script was run that automates dipping the strips into the liquid samples, switching lighting, and recording time-lapse images on all test strips simultaneously for the indicated time period. The images were transferred to an external computer for subsequent analysis of capillary rise using ImageJ software (NIH, USA^45^) (Fig. 1c). Instantaneous fluid velocity was determined at multiple timepoints by determining change in height between pairs of images, and calculated as change in height divided by change in time.

### 2.5. Flow dynamics of activated blood

To establish proof-of-concept for measuring the dynamics of coagulation during capillary flow, 100 mm long test strips were loaded by freeze-drying with 0, 5, 15, 50 and 150 U mL^−1^ thrombin in water. Time-lapse images were taken at frame rate of 6 full-resolution images per second, and the height of blood *H*(*t*) sample rising in the microcapillaries analysed by using ImageJ, allowing estimation of instantaneous mean superficial fluid velocity, d*H*/d*t*. Replica tests were performed with 5 different donors, with a similar response to the range of thrombin doses tested seen each time.

### 2.6. Analytical modelling of fluid rise in the microcapillaries.

Although it is known that blood shows a complex rheology, with viscosity dependent on shear rate, we noticed that the initial stages of capillary rise in our vertical microfluidic strips resulted in mean superficial velocities of 10–30 mm s^−1^ corresponding to theoretical wall shear rates of 100–600 s^−1^, similar to the wall shear rates observed in blood vessels in human body. Under those conditions, viscosity of blood can be assumed to be independent on shear rate. Therefore, we modelled the capillary rise of sample fluids in vertical hydrophilic microfluidic strips having a wide range of fluid properties using a pressure balance model which enabled capture of both dynamic flow and endpoint equilibrium of the liquid samples drawn into microcapillaries by capillary action. Fluid flow in a vertical capillary occurs *via* a balance between the Laplace pressure, which drives the fluid using capillary action, and frictional and gravitational resistances which both increase as the liquid rises vertically. The Laplace pressure drop (Δ*P*_L_) can be defined as in eqn (1):1
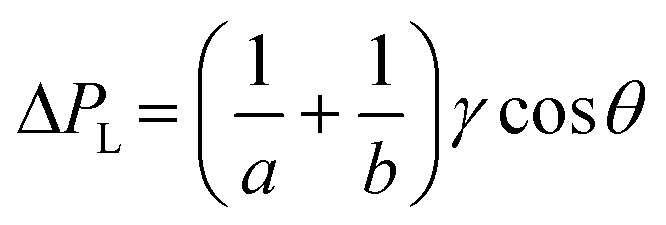
where *γ* is liquid–air surface tension, *θ* is liquid–wall contact angle, and *a* and *b* are, respectively the major axis and minor axis for the elliptical microcapillaries. The hydraulic diameter, *d*_h_ can be calculated using the cross section, *A*, and perimeter, *P* for each individual microcapillary, with *d*_h_ = 4*A*/*P*. Frictional resistance-imposed pressure drop (Δ*P*_F_) is given by the Darcy–Weisbach equation shown in eqn (2):2
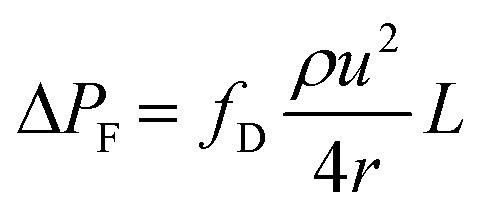
where *u* is mean superficial fluid velocity and *L* is the total fluid length within the microcapillary. For laminar flow, the Darcy friction factor *f*_D_ is given in eqn (3):3
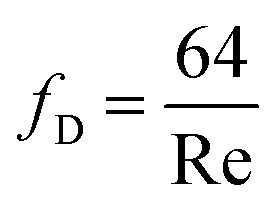
where Reynolds number, Re, is shown in eqn (4):4
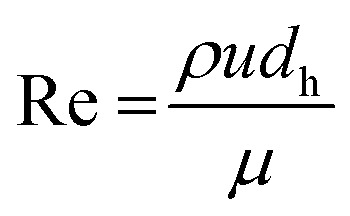
where *ρ* is fluid density and *μ* is dynamic fluid viscosity, which has been assumed as constant and independent of shear rate (only realistic for Newtonian fluid and non-Newtonian fluids like blood at higher shear rates). The liquid pressure head (Δ*P*_H_) for a liquid height *H* (measured between the liquid level in sample reservoir and the meniscus) is given in eqn (5):

5 Δ*P*_H_ = *ρgH*

where *g* is gravitational acceleration. A full pressure balance can be defined as in eqn (6) and solved to obtain the instantaneous fluid rise velocity, *u*(*t*) using eqn (7):6
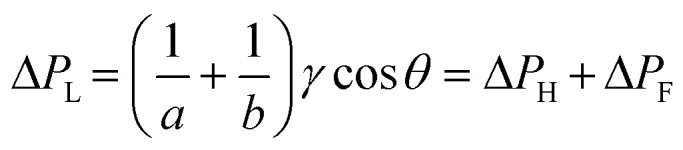
7
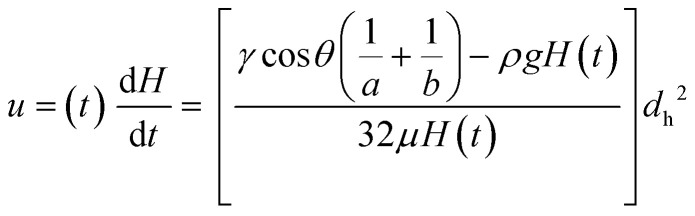
Rearranging, a differential equation can be yielded relating instantaneous fluid rise in the capillaries with the fluid properties, *ρ* and *μ*, interfacial forces/properties, *γ* and *θ*, and inner dimension of the microcapillary:8
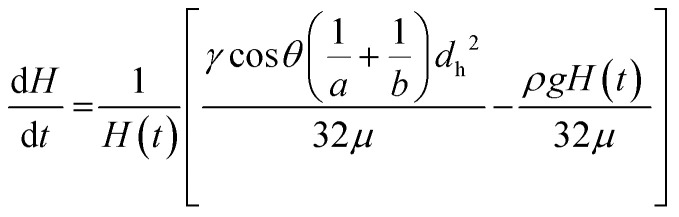
For equilibrium capillary rise, when Δ*P*_L_ equals Δ*P*_H_, the equilibrium fluid height, *H*_eq_ can be determined using:9
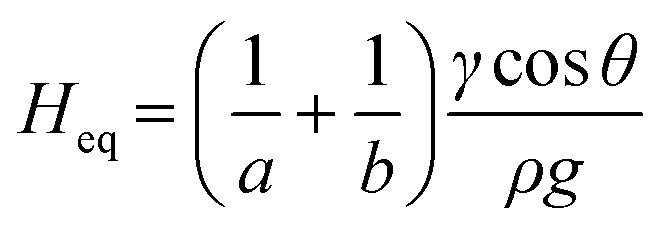
As shown in eqn (9) equilibrium capillary rise is independent of fluid viscosity as there are no flow resistances present at equilibrium point, *i.e.* after flow has stopped.

## 3. Results and discussion

### 3.1. Calibration of endpoint and dynamic capillary rise with fluid properties using glycerol–water mixtures

**Fig. 2:**
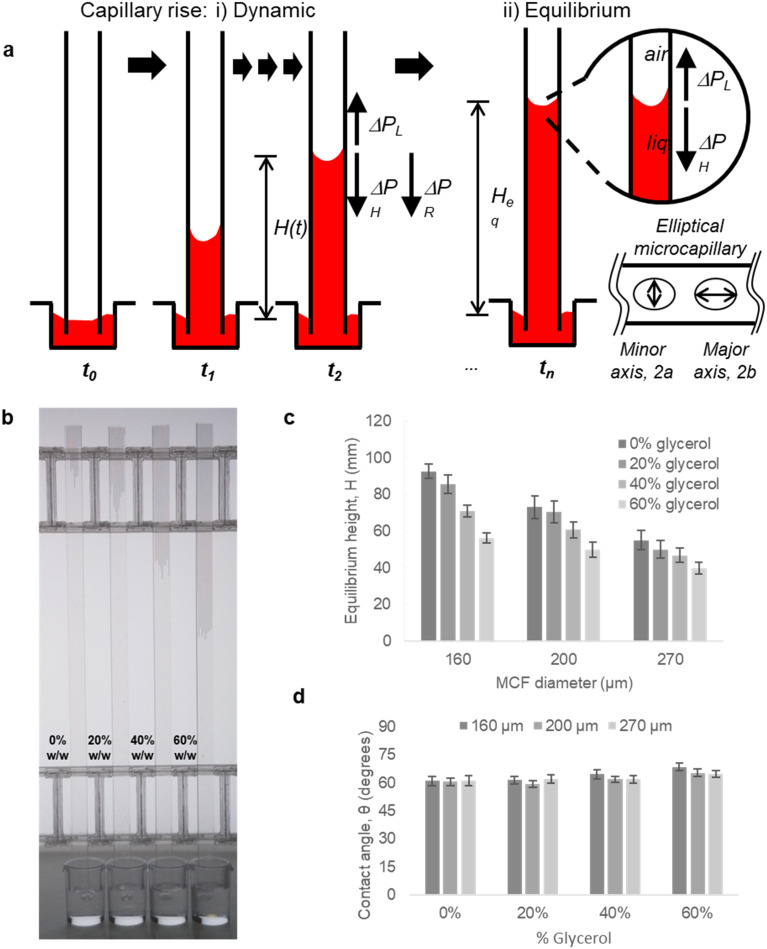
Capillary flow in vertical ‘dip stick’ MCF strips. (a) Liquid rise in microcapillaries was modelled by considering a full pressure balance to each individual capillary. The transient (dynamic) (i) movement of fluid resulting from a balance between Laplace force, hydraulic liquid height and liquid flow resistance, is highly dependent on the viscosity and density of fluid. Once liquid reaches equilibrium height (ii) (right hand side), the Laplace pressure balances out the liquid height pressure, which is only dependent on the density of the fluid, thus equilibrium endpoint measurement does not measure viscosity. (b) As glycerol concentration increases, the equilibrium height decreased, showing equilibrium height reveals differences in fluid density. Image shown for 160 μm diameter microcapillaries. (c) Equilibrium liquid rise in microcapillaries for glycerol–water mixtures in microcapillary strips with different inner diameters, shows capillary rise is more sensitive to differences in fluid density for smaller diameters. Error bars indicate one standard deviation from 10 individual microcapillaries. (d) Mean contact angles, *θ* estimated for individual MCF strips (sets of 10 microcapillaries) showing *θ* is independent of the density of glycerol–water mixtures and inner diameter of microcapillary.

To fully demonstrate how our technique based on both endpoint equilibrium, and dynamic capillary rise in the vertical ‘dip stick’ MCF strips is sensitive to fluid properties, we carried out simple validation experiments using Newtonian fluids of known viscosity, density, and surface tension. Test strips were robotically dipped into glycerol–water mixtures with glycerol concentrations ranging 0–60% w/w (Fig. 2) with a wide range of known viscosities and density (Table S2†) and capillary rise recorded by time lapse imaging. We observed the equilibrium capillary height, given by eqn (9), decreased with the increase in glycerol concentration due to a reduction in the surface tension and increase in fluid density. We imaged microfluidic strips with different inner diameters of 156.3 ± 14.63, 201.6 ± 11.90 and 261.2 ± 29.60 μm (Table S1†), to confirm that equilibrium height reduces as diameter increases (Fig. 2b and c). This follows eqn (9), with *H*_eq_ proportional to the reciprocal of diameter. As a quality check, estimates of the equilibrium contact angle *θ* based on known fluid properties and observed *H*_eq_ values were consistent across all microcapillary diameters (Fig. 2d), with *θ =* 61.0 ± 0.36, for pure water (*i.e.* 0% w/w glycerol) and *θ =* 62.8 ± 2.57 across all diameters. As wall material and coating procedure were similar regardless of inner diameter, it was reassuring that derived surface properties were consistent.

Alongside determining endpoint equilibrium, the microfluidic imaging system enabled tracking the transient vertical flow of fluids up the ‘dip stick’ test strips from time-lapse images, streamlining image analysis and allowing us to determine dynamic parameters that cannot be derived from equilibrium or endpoint observation. A full pressure balance using upward Laplace forces balanced by hydrostatic head and frictional resistance forces (eqn (6) and (7)) was applied to model the liquid rise in the hydrophilic microcapillaries (Fig. 2a), taking inspiration from our earlier work with the ‘Lab on a stick’ concept.^24^ We note this model is simplified by excluding an inertial term and assumes that instantaneous velocity primarily reflects a net pressure balance, with minimal contribution by acceleration. For capturing more accurately capillary forces and considering the elliptical shape of the microcapillaries, the minor and major axis of each individual capillary in the 10-bore MCF, having average diameters of 160, 200 and 270 μm, were used to take into consideration the elliptical shape of the microcapillaries. Due to the manufacturing melt-extrusion process, the middle eight capillaries were slightly wider than the outer two, and the axes narrowed moving from the middle to the edges resulting in minor deviations in the flow of liquid (Table S1†).

Time-resolved imaging of glycerol–water mixtures allowed instantaneous superficial fluid velocity, *u*(*t*) given as d*H*/d*t* to be recorded and then plotted *versus* time or reciprocal of *H*(*t*), which showed that as expected (eqn (8)) the dynamics of fluid rise in the vertical microcapillary films is also very sensitive to the fluid properties (Fig. S2†). We note that as predicted by eqn (8), a largely linear relationship was seen when instantaneous velocity was plotted against 1/*H*(*t*), and we found this plot to be a valuable way to visualise the vertical flow dynamics, alongside plotting height *vs.* time. In contrast to the equilibrium rise, this linear relationship between velocity and reciprocal height was independent of capillary diameter. The pressure balance model was validated by modelling flow of glycerol–water samples with known properties using excel solver, from which our observed and derived values gave excellent prediction of the d*H*/d*t* (Fig. S2†) with experimental data confirming our models. A full comparison of predicted and experimentally measured viscosity, surface tension, and contact angle suggests these values for the smaller two capillary diameters stayed within ∼13% and even for the largest capillaries stayed within 20%.

### 3.2. Capturing properties of blood and blood components during capillary flow

**Fig. 3:**
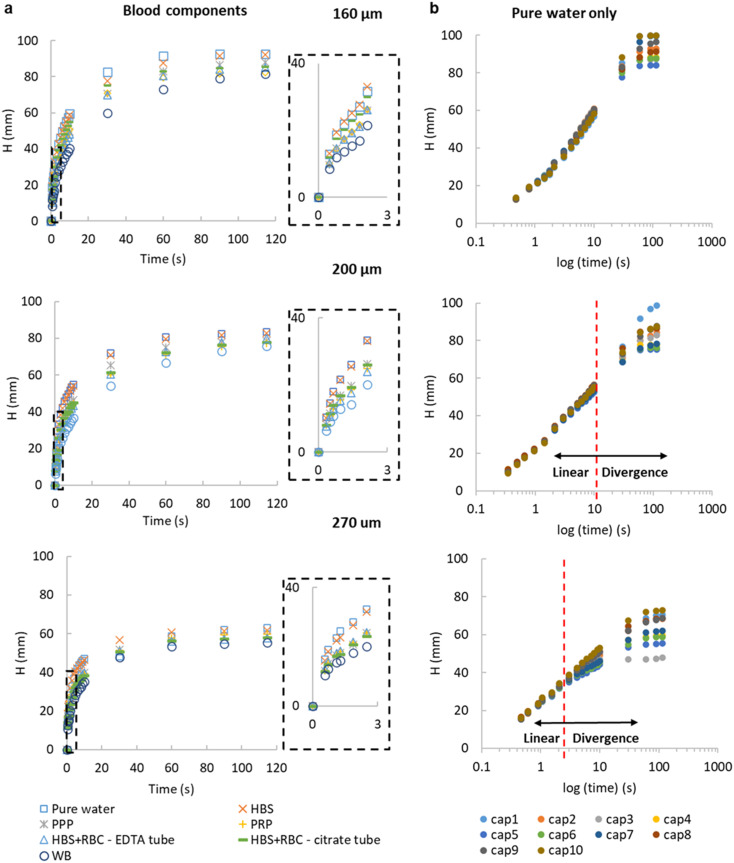
Instantaneous vertical capillary rise with a range of liquid samples. (a) Net capillary rise *H*(*t*) *versus* time for water, buffer (HEPES buffered saline HBS), plasma (platelet rich plasma PRP and platelet poor plasma PPP), washed red blood cells collected with different anticoagulants (RBC) and whole blood (WB), all tested in ‘dip stick’ MCF strips of inner diameter 160, 200 and 270 μm. The first 3 seconds of capillary rise (insets) show the fastest vertical superficial fluid velocity, and the largest difference between blood components and water. Data points indicate 10 replicate capillaries. (b) Flow of water in 10 individual capillaries. Plotting height against time on a log *x* axis shows initial fluid velocity (shown for pure water) virtually independent of the inner diameter of the microcapillary, therefore different microcapillaries within the same strips and also different inner diameters show similar initial slope. The semi-log plot illustrates two phases of flow, with divergence at later timepoints showing that the impact of gravity increases for larger diameters. Similar results were observed with two repeats with 160 and 270 μm strips, and three repeats with 200 μm.

With the working principle of the technique established for Newtonian glycerol–water fluid mixtures, transient fluid velocity and endpoint analysis were performed with different liquids, comparing water and buffered saline to non-Newtonian fluids relevant to blood analysis, *i.e.* whole blood and separated blood components – both plasma and washed red cells. This covered known density and viscosity properties of water, buffer and blood components (Table S3†). The instantaneous fluid rise velocity in the microcapillaries (Fig. 3a) fell rapidly over 30 seconds for all the conditions with the fastest linear fluid velocity (∼10–20 mm s^−1^) occurring within the first 3 seconds of capillary rise (see inset plots in Fig. 3a). The pure water and HBS samples showed very similar superficial fluid velocity initially around 15–30 mm s^−1^ then rapidly falling below 10 mm s^−1^ after reaching 25 mm height (*i.e.* 1/*H*(*t*) below 0.04). Both plasma and red cell components of blood had slower d*H*/d*t* than water or buffer, initially 10–20 mm s^−1^, indicating higher viscosity, in line with pressure balance model in eqn (8). Whole blood showed the slowest initial velocities, starting at 5–15 mm s^−1^ then falling to ∼2.5 mm s^−1^ by 25 mm height rise. We estimated mean wall shear rate, *γ*_w_ to stay in the range of 100–800 s^−1^, based on *γ*_w_ = 8*u*/*d*_h_, for this range of superficial fluid velocities and inner microcapillary diameters, matching the range of wall shear rates experienced within blood vessels in the human body. Similar d*H*/d*t* profiles were seen for platelet-rich and platelet-poor plasma samples; similarly, both RBC suspensions showed similar flow profiles regardless of anticoagulant used for blood collection; in both case these both plasma rich and red blood cells suspension individually showed higher instantaneous superficial velocity and higher equilibrium heights compared to whole blood, suggesting that these two blood components contribute additively to increase in dynamic fluid viscosity, reflected in reduced fluid velocity and lower heights at all times (Fig. 3). As expected, both d*H*/d*t* and *H*_eq_ values were lower for larger diameter microcapillaries (Fig. 3), which is again in agreement with pressure balance model in eqn (8). Note that blood is a complex fluid with viscosity dependent on shear rate, yet for the range of shear rates experienced in initial stage of capillary rise it is a fair approximation to consider dynamic viscosity of blood as constant based on evidence gathered from literature.^25–27^

By studying the initial gradient of superficial fluid velocity, d*H*/d*t* over hydraulic height *H*(*t*), we noticed that the larger diameters presented a greater variation in linear fluid movement across the 10 capillaries in each microfluidic strip, whereas smaller diameters were more consistent, shown by the smallest spread in datapoints on the right-hand side of Fig. 3b, corresponding to initial time points. This is presumably related to small variations in Laplace pressure, with wetting of the inner hydrophilic capillary becoming more inconsistent as inner capillary diameter increased. The initial superficial fluid flow revealed largely independent of internal diameter, in line with observations for glycerol : water mixtures (Fig. S2†), in the case of more complex samples divergence from linear flow occurred earlier on for larger diameters (Fig. 3b). The ability of our technique and imaging system to distinguish small difference in microcapillary flow for blood and blood components illustrates the importance of having fast time-lapse capture of fluid velocity data especially from early time points. The equilibrium, or single endpoint measurements alone provided limited data compared to considering transient flow, as velocity is strongly dependent of viscosity and frictional resistances. This indicates that the time-resolved robotic imaging instrument improves our ability to measure important fluid properties such as viscosity *vs.* a single endpoint or equilibrium image. Also, it is worthwhile mentioning that our method is not able to provide comprehensive rheological measurements nor replacing advanced rheology equipment including microcapillary rheometers for lacking consideration of shear dependence of viscosity; however, the simplicity of operation and simple instrumentation compensates for this.

**Fig. 4:**
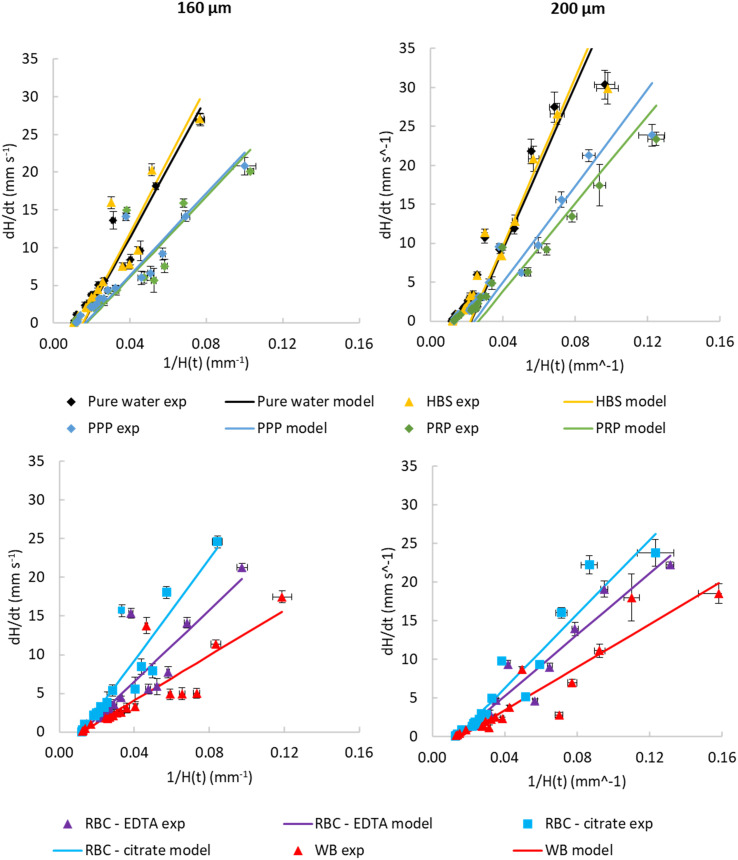
Observed dynamic flow of blood and blood components is consistent with higher viscosity than water. Comparison of experimental instantaneous superficial fluid velocity d*H*/d*t vs.* reciprocal *H*(*t*) with modelled velocities calculated from transient pressure balance. Data shown comparing water and buffer with PPP and PRP (top) and comparing WB with washed RBCs for both 160 and 200 μm inner diameter. Parameters used for models, and constraints used to fit these parameters, are presented in Table 1. These illustrate that the model reasonably fits experimental data. Data indicates 10 replicate capillaries where error bars indicate ± SD from a single donor; similar results were observed in 3 repeat experiments with different donors.

The pressure balance model predicts a linear relationship between d*H*/d*t* velocity and reciprocal of liquid head, 1/*H*(*t*) for the initial stage of capillary rise. This is the stage at which viscosity of blood can be safely assumed to be constant and independent of shear rate based on data widely available in literature. The non-linear term in eqn (8) disappears when *H*(*t*) → 0, therefore we explored how closely model predictions matched the experimental data for blood components. Initial velocities depended significantly on sample properties (Fig. 4), with the blood and blood components having higher viscosity than buffer or water, translating in reduced d*H*/d*t* values. Again, time-resolved imaging provided significant additional data on viscosity and frictional resistances that could not be gained from equilibrium, endpoint analysis, with most data sets ending at similar endpoint value of 1/*H*(*t*) in *x* axis. A close fit of the capillary flow to the pressure balance model (both dynamic and endpoint) allowed extracting multiple unknown parameters, in particular fluid properties but also air–liquid–wall interface properties. The steady, shear-rate independent model worked well for 160 and 200 μm microcapillaries but with the larger 270 μm, we noticed some degree of divergence from linearity (Fig. S4†). This is presumably linked to the Fahraeus–Lindqvist effect,^47^ with blood viscosity decreasing with decreasing channel size^28^ during *in vivo* flow studies of thrombosis. This suggests our approximation of shear-independent blood viscosity in early time points is more realistic and our new technique can be more effective when narrow microcapillaries are used. Linked to higher Laplace forces, narrower capillaries also offer longer overall distance travelled by the fluid potentially providing higher resolution measurements with the same camera setup.

The highest fluid velocities were observed at very early stages of capillary flow, estimated from images captured within the first 3 seconds at very short intervals. It must be noted that we expected greater noise in these instantaneous velocities, where time intervals are very short and small variations in image capture timing and/or dipping of the strip will result in larger noise in estimated velocity compared to linear predicted velocities. We believe the apparent higher noise at observed velocities above 5 mm s^−1^ (*e.g.*Fig. 4) can be accounted for by the our lower confidence in time differences between images at very early stages, compared to the longer time intervals between images used to estimate velocity at later times when velocity is far lower. This apparent noise in instantaneous velocity at early timepoints contrasts to the observation that velocities become less dependent on capillary diameter (Fig. 3b), and variance between replicate capillaries is low. This explanation can be tested using additional image capture methods such as high-resolution, high-framerate video cameras. Alternatively, the pressure-balance model assumes ‘quasi-steady state’ and therefore neglecting fluid acceleration or consider fluid inertia, that could potentially affect very early time points where initial acceleration of liquid into the capillaries takes place, which might also contribute to variation between observed and predicted velocities.

Table 1 lists the fitted model parameter values which set constraints reflecting the expected range of these properties for blood and blood components, to improve how readily the derived parameters could be determined empirically. We also fixed density to reduce the number of unknown parameters, using well characterised density values for blood and plasma taken from the literature (summarised in Table S4†). For glycerol : water mixtures we used surface tension values from the literature to calculate contact angle from equilibrium rise. However, for blood components, we combined the surface properties affecting Laplace pressure at the capillary meniscus, *i.e.*, surface tension and contact angle, into a single term, cos(*θ*) × *γ*, in order to minimise subjectivity of the calculations.

**Table 1:** Fluid parameters estimated to fit the transient/dynamic pressure balance model with flow dynamics observed experimentally, and used in comparison between observed and modelled velocities plotted in Fig. 4

	160 μm		200 μm		Constraints
	cos *θ*·*γ* (mN per degrees per m^2^)	Dynamic viscosity, *μ* (cP)	cos *θ*·*γ* (mN per degrees m^2^)	Dynamic viscosity, *μ* (cP)	Dynamic viscosity (cP)
Water	0.025	1.04	0.022	1.04	1.04
HBS	0.025	1.04	0.022	1.04	1.04
PPP	0.023	1.70	0.021	1.70	<1.7
PRP	0.024	1.70	0.019	1.70	<1.7
RBC–EDTA	0.034	2.86	0.037	4.72	>2, <6
RBC–citrate	0.034	2.02	0.036	3.79	>2, <6
WB	0.034	4.58	0.033	6.00	>2, <6

Our experimental results showed that the fluid rise in the capillaries can be reliably predicted with the pressure balance modelling (Fig. 4). The similarity between water and buffer, and between plasma with or without platelets, provides reassurance that these observations are repeatable. Some deviations from the model were seen with the highest velocities, which is expected considering these were estimated from sequential images only a fraction of a second apart, and with high velocities meaning relatively small variations are amplified. This shows that time- and distance-resolved imaging allows us to determine not only endpoint equilibrium height data based on total migration distance, but also derive viscosity and frictional resistances by studying transient flow at early time points. Thus, the open source RMS instrument improves microfluidic blood analysis allowing unknown liquid properties such as density and viscosity can be derived.

### 3.3. Capturing changes in capillary flow dynamics for stimulated blood following activation by thrombin

To demonstrate the ability of our microfluidic and imaging technique for capturing changes in coagulation and fluid properties, we measured flow dynamics of blood following stimulation by thrombin, an enzyme that triggers the haemostatic response through both platelet activation and coagulation *via* the cleavage of fibrinogen to fibrin.

**Fig. 5:**
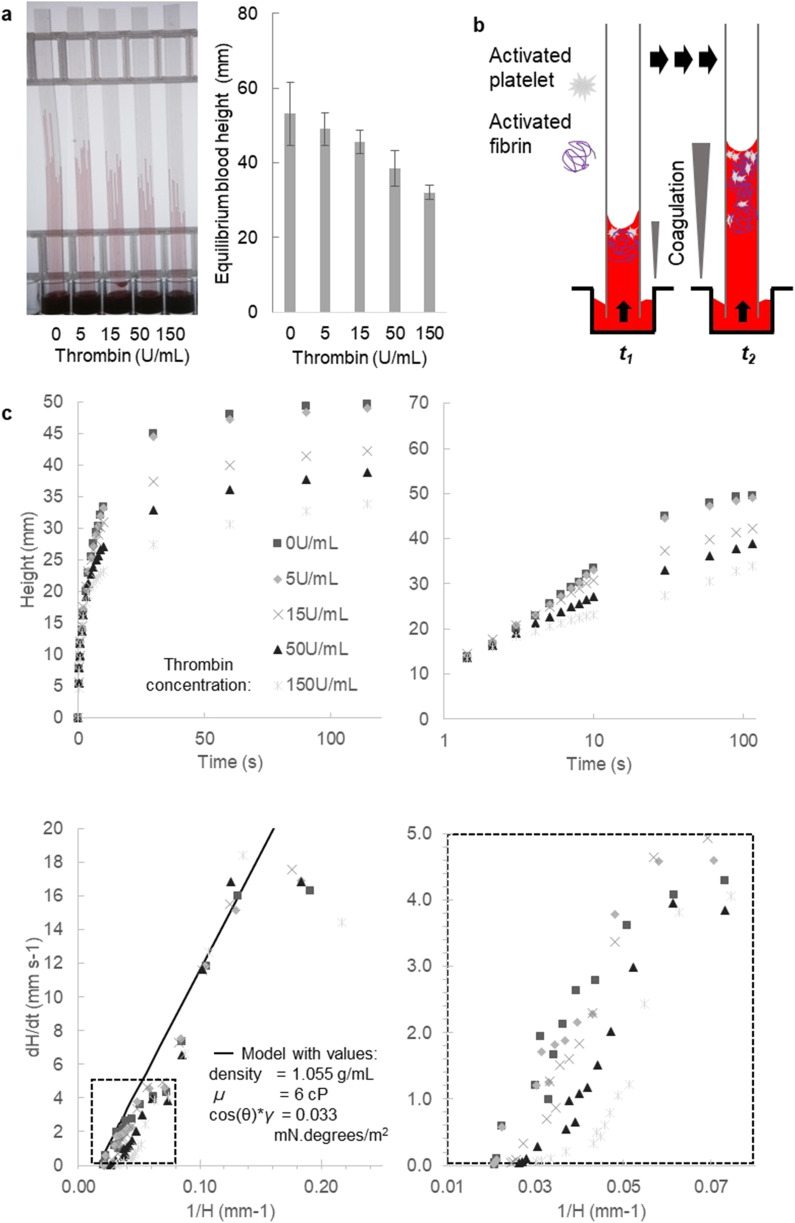
Stimulation of blood samples with thrombin rapidly modifies the dynamics of microcapillary flow. (a) The overall rise of blood at fixed timepoints was consistently sensitive to the amount of thrombin loaded within microcapillaries (loaded with 0, 5, 15, 50 and 150 U mL^−1^). Mean fixed-time endpoint heights of 50 replicate capillaries were plotted from 5 different 10-bore MCF strips, each strip tested with blood from a different donor; error bars indicate one standard deviation. (b) Expected change in coagulation over time and distance along capillary, as fibrin forms and platelets aggregate, with greatest coagulation expected near the meniscus. (c) Flow dynamics and equilibrium capillary rise changed with thrombin concentration, with data shown for the mean of the 10 replicate capillaries from a 10-bore MCF strip for one donor for clarity; similar profiles were observed in independent repeat experiments with blood from 5 different donors. The four different plots illustrate how distance and velocity are affected by triggering coagulation with thrombin.

Stimulating whole blood with thrombin within the capillaries led to a rapid and concentration-dependent reduction in the height of capillary rise at all timepoints, compared to unstimulated blood. When multiple replicate endpoint measurements were taken to estimate this overall retardation of vertical flow, similar responses were found with multiple donors (Fig. 5 and S3†). Note that the concentration of thrombin loaded into the microcapillaries during freeze drying, appear somewhat higher than those typically used for thrombin stimulation in other studies.^48^ During rapid capillary rise of the blood/sample, there is stimulus dissolution and mixing taking place as well as fluid movement. At this stage of development of this technique, it is not possible to establish the actual concentration of thrombin experienced in the blood that is being released; this may account for the higher loading dose required to get stronger observed responses.

We would expect viscosity to increase over time following thrombin dissolution, and furthermore a gradient of coagulation can be expected with highest viscosity at the top where the blood has been exposed to stimuli for longest (Fig. 5b). Furthermore, for simplicity, at this initial stage of analysis any impact of coagulation localised near the meniscus on surface properties (surface tension and contact angle) was not considered. With stimulation occurring within the microcapillaries during flow, it is not possible to determine all surface properties experimentally from the height at equilibrium, in contrast to unstimulated liquids, which do not change during capillary rise (*e.g.*Fig. 2–4). This is for two reasons: firstly, the equilibrium height will only reflect the surface properties at that timepoint, which may differ from earlier times; secondly, stimulation eventually leads to coagulation to a point when flow ceases as a cross-linked fibrin gel is formed, so equilibrium may never be reached. To confirm those assumptions, we laid test strips flat after 30 minutes but with thrombin-loaded capillaries found no further flow could occur (data not shown), confirming that thrombin stimulation eventually leads to capillary blockage.

Nevertheless, we clearly observed decreased fluid velocity that would be consistent with a rapid and dynamic increase in viscosity following stimulation, seen most clearly when instantaneous d*H*/d*t* was plotted against reciprocal height 1/*H*(*t*), as expected since coagulation of blood during haemostasis should lead to obstructed flow (Fig. 5c). This effect was reproducible with two further blood donors showing similar response to thrombin (Fig. S3†) demonstrating that coagulation can be activated within the capillary during capillary rise, and that instantaneous velocity is sensitive to rapid changes in coagulation. In future, higher resolution imaging should be able to directly observe the meniscus curvature, which would allow any changes in curvature to be monitored during dynamic stimulation. This additional information could enable more direct estimation of viscosity changes, by monitoring changes in instantaneous surface properties. However, there would be a trade-off between using far higher resolution cameras (thus more expensive instrumentation), or a smaller field of view (thus fewer samples imaged), to simultaneously collect information about meniscus position (to monitor instantaneous velocity) and curvature (to monitor surface properties).

**Fig. 6:**
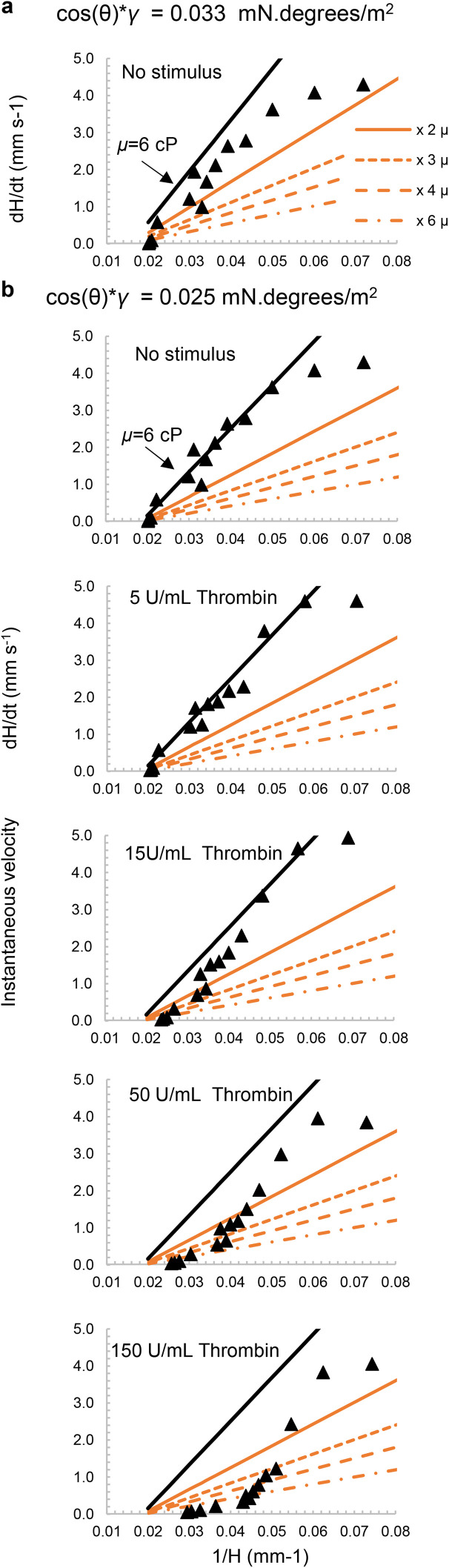
Modelling viscosity changes following thrombin stimulation of blood. The velocity of blood flow into capillaries was modelled using known blood density (1.055 g mL^−1^) plus surface property value (cos(*θ*) × *γ*) derived from the data, as follows: (a) no stimulus with cos(*θ*) × *γ* = 0.033 mN per degrees per m^2^) as derived for whole blood in Fig. 5; (b) no stimulus and with thrombin (5, 15, 50 and 150 U mL^−1^) with adjusted lower surface property value that fits the no stimulus data better *i.e.* cos(*θ*) × *γ* = 0.025 mN per degrees per m^2^). The mustard-coloured lines represent increases in viscosity ranging from 2×, 3×, 4×, to 6× the initial viscosity whilst keeping the surface property value constant. In all cases, the indicated range of viscosities are plotted to explore how well the pressure balance model could estimate the impact of increased viscosity over time following stimulation. Predicted velocity dynamics fitted experimental data earlier and with higher viscosities as thrombin concentrations increased; in contrast without stimulus the model fits observed values closest with no change in viscosity.

Our current pressure balance model does not consider variation of fluid properties over time or distance, and as noted above cannot distinguish between changes in viscosity (that affect resistance to flow) and alterations in surface properties (that affect the pressure drop that drives flow). In spite of these limitations, we explored if the observed data was consistent with an increase in viscosity over time after stimulation simply by plotting modelled instantaneous velocities for a series of higher viscosities, keeping all other parameters fixed (Fig. 6). Fig. 6a shows our model fitted to blood flow without a stimulus using the surface property value determined from Fig. 5; we found surface properties were somewhat changed after freeze-drying the stimulus, indicated by a better fit with a lower surface property value (Fig. 6b). For strips with stimulation, by plotting a range of theoretical viscosity increases from 2×, 3×, 4×, and 6× the starting viscosity, we observed a shift of the d*H*/d*t versus* 1/*H* data that matched predictions of lower starting viscosity at earliest times (*i.e.*, higher 1/*H*), downwards towards fluid flow at higher viscosities at later times. This effect increased and took place earlier as the loaded thrombin concentration increased (Fig. 6b) consistent with a concentration- and time-dependent viscosity increase following stimulation during vertical capillary flow. This suggests our technique has the potential to capture dynamic changes in blood coagulation during capillary flow from observation of instantaneous velocity, this cannot be matched by any of the techniques currently available, not even by rheological measurements as coagulation is a fast process making measurement of transient properties especially challenging. Whilst thrombin stimulation was used here as proof-of-concept, this assay could be used with other stimuli that trigger coagulation and/or platelet activation. The use of whole blood means that both fibrin formation and platelet activation are triggered simultaneously, more closely mimicking physiological conditions. Such a test is most likely to be used for routine haemostatic tests, similar to those administered before surgical procedures or to investigate the suitability of an anticoagulant drug for treatment. The main benefit over existing coagulation tests is the large number of replicates possible in a single 30 second test, permitting a wider detection range, allowing either multiple samples, or expansion to a larger range of stimuli, to be tested in parallel. The ability to measure 120 capillaries simultaneously opens the door to larger scale stimulation panels beyond our proof-of-concept with thrombin.

Measurement of migration distance has previously been used in horizontal flow by adding stimulated WB to a microfluidic chip.^22,23^ The stimulated blood reduced migration distance, indicating increased platelet aggregation induced by a combination of two stimuli, ADP and collagen. The new imaging system not only determines the final migration ratio but also captured the instantaneous velocity of the fluid by taking at least 6 frames per second. Changes in velocity have also been observed with other tests for example those analysing the impact of platelet activation. Velocity of WB in microchannels was slower in the presence of ADP than without,^49^ this concurred with our thrombin activated WB, as we observed decreasing velocity over time due to increased coagulated along the capillary length, lower exposure to the thrombin stimulus and which lowered the equilibrium heights.

Previous studies used microfluidic devices which had no more than 2 channels, and measure flow in horizontal rather than vertical flow.^20–23,49^ The use of 2 channels is typically used to compare migration distance between stimulated *vs.* unstimulated blood, yet the use of a single stimulation significantly reduces the amount of information generated, especially since biological systems are often better quantified by concentration–response relationships. While here we used 10 replicate capillaries per condition to demonstrate the concept, future analysis combining ‘dip stick’ devices with the RMS instrument is expected to make full use of 12 simultaneous test strips containing up to 10 conditions per strip, yet minimising the blood volume required. Furthermore, in contrast to laboratory instruments such as flow cytometers, our integrated microfluidic imaging system can be used without training, with illumination, dipping, and imaging all automated. The low cost (∼100× cheaper than flow cytometer) and open source hardware make this system accessible to anyone.

A challenge that remains unaddressed with our technique is the difficulty of loading reagents in the microcapillaries without risking altering the capillary flow. Flow in capillaries is strongly influenced by the surface properties within the capillary. Whilst thrombin freeze-dried within capillaries clearly activates blood, our initial model fitting suggested the loaded capillaries may also have altered surface properties (Fig. 6). This could either be due to coagulation triggered by thrombin near the meniscus, or to the loaded thrombin affecting contact angle and/or surface tension. We noted higher noise between replicate capillaries when using freeze-dried test strips (regardless of the loading reagent) than when validating the method using strips without reagent freeze-drying (*e.g.* compare Fig. 5a*vs.*2c and d). This loading process is a major focus of ongoing study and optimisation, especially to expand the range of stimuli to make full use of the high sample capacity of the system.

## 4. Conclusions

Our imaging technique integrating hydrophilic microfluidic strips with time- and distance-resolved imaging with open-source instrumentation demonstrates it is possible to capture coagulation and changes in blood properties in disposable microfluidic devices, as coagulation happens within the microcapillaries, at shear rates similar to those found in blood vessels in human body. Fitting of experimental capillary rise data at early stages of capillary rise with simple pressure-balance modelling enables quick derivation of vital fluid properties from rapid experiments using extremely simple capillary microdevices. This approach and system has the capacity to significantly improve accessibility and quality of blood coagulation data that can be derived from analysis of coagulation components in blood, by exploiting simple, mass-produced microfluidic devices, paving the way towards large-scale population measurements of vital blood properties. Our methodology offers rapid quantitative testing, which can be completed in less than 2 minutes. Time-lapse imaging allows recording of valuable information, which occurs within the first 2–3 seconds of the rise, when compared to other studies based solely on the endpoint data. The synergistic combination of open-source instrumentation with affordable microfluidics offers a pathway to higher throughput blood agglutination testing without the need for expensive laboratory automation, paving the way to a global point-of-care haemostasis testing essential for rational prescription of anti-coagulation or anti-platelet drugs.

## Ethical approval

All experiments were performed with informed written consent obtained from all human subjects in accordance with the Declaration of Helsinki and with the approval by the University of Reading Research Ethics Committee (Reference Code: UREC 20/20).

## Conflicts of interest

A. D. Edwards and N. M. Reis are the inventors of patent application protecting aspects of the novel microfluidic devices tested in this study and is a director and shareholder in Capillary Film Technology Ltd, a company holding a commercial license to this patent application: WO2016012778 “Capillary assay device with internal hydrophilic coating” inventors AD Edwards, NM Reis.

## Supplementary Material

SD-002-D3SD00162H-s001
