# Supplementary material for: Time- and distance-resolved robotic imaging of fluid flow in vertical microfluidic strips: a new technique for quantitative, multiparameter measurement of global haemostasis
Source: Sens Diagn. 2023 Oct 17;2(6):1623–37. doi: 10.1039/d3sd00162h (PMC10633108; doi:10.1039/d3sd00162h)
Supplement: SD-002-D3SD00162H-s001 [file SD-002-D3SD00162H-s001.pdf]

Electronic Supplementary Material (ESI) for Sensors & Diagnostics.
This journal is © The Royal Society of Chemistry 2023

Time- and distance-resolved robotic imaging of fluid flow in vertical microfluidic strips: a new technique for quantitative, multiparameter measurement of global haemostasis

Rüya Meltem Sarıyer[a], Kirandeep Gill[a,b], Sarah H. Needs[a], Daniel Hodge[c], Nuno M. Reis[b], Chris I. Jones[c], Alexander D. Edwards[a*]

[a]Reading School of Pharmacy, University of Reading, Whiteknights, Reading, RG6 6UB, United Kingdom. r.sariyer@pgr.reading.ac.uk, kkgill@uvic.ca, s.h.needs@reading.ac.uk

[b]Department of Chemical Engineering and Centre for Biosensors, Bioelectronics and Biodevices (C3Bio), University of Bath, Bath BA2 7AY, United Kingdom. n.m.reis@bath.ac.uk

[c]Reading School of Biological Sciences, University of Reading, Whiteknights, Reading, United Kingdom. c.i.jones@reading.ac.uk, d.j.hodge@pgr.reading.ac.uk

*Corresponding author contact a.d.edwards@reading.ac.uk +44 (0) 118 378 4253

This document describes the development and working principle of the robotic microfluidic imaging system (RMS) (Section S1), provides detailed methodology for the preparation of Microcapillary Film test strips (S2) and includes figures and tables in addition to the main text (S3).

Contents

Time- and distance-resolved robotic imaging of fluid flow in vertical microfluidic strips: a new technique for quantitative, point-of-need measurement of blood coagulation 1

S1 Development and working principle of the RMS 1

S2 Preparation of MCF test strips 3

S3 Supplemental tables and figures 4

Supplementary References..........8

S1 Development and working principle of the RMS

By following the principles of free open-source hardware (FOSH) we provide a complete bill of materials (BOM), technical and user instructions and 3D CAD designs for anyone to build or adapt the device, through a design repository hosted on GitLab and via a permanent digital object identifier (DOI:10.5281/zenodo.6617301) on the Zenodo data hosting site. We developed a low-cost (approximately £300 at 2022 prices), open-source, customizable robotic microfluidic blood analysis system to scale up measurement of blood coagulation and platelet function, named the Robotic Microfluidic imaging System (RMS). The RMS system is based on the automatic control of every part of the system (camera, servo motor and lightbox) by a Raspberry Pi computer. A simple aluminium extrusion frame coupled with bespoke 3D printed parts combines all necessary functions in a small footprint (350 mm x 415 mm x 288mm).

The test method allows high-throughput microfluidic testing using multiple devices in “dip-stick” format, using the “Lab-on-a-Stick” method to multiplex reagent mixing with sample [1]. Hydrophilic

microcapillary films (MCFs) loaded with coagulation-activating stimuli are used as test strips. Microfluidic technology combined with RMS allows 120 tests to be performed simultaneously by 10 capillaries within each of 12 different test strips in a single automated experiment. Kinetic data of capillary flow velocities within these inexpensive microfluidic devices is gained by capturing 6 images per second after the test is stated by robotic sample dipping. This transforms simple distance-based capillary flow assays from endpoint (providing limited information about dynamics of flow and viscosity) to detailed analysis of the dynamics of rapidly changing blood properties following activation with a coagulation stimulus. Information including the material list, 3D designs files, and software codes for building and using the RMS system have been published with open-source hardware licenses, allowing anyone to build and use the system, or to customise to different applications that benefit from robotic digital imaging.

A LED lightbox is turned on by controlling a relay, after that, a servo motor via the gantry plate and wheels is dipped the MCF strips holder into the blood sample which moves into the capillaries by capillary action and after the test completed the holder is returned, and the LED is turned off. In the meantime, a Raspberry Pi camera V2.1 takes time-lapse images during the experiment. This camera can take between 6 images within 1 second. To be able to take 6 images in 1 second, the number of images to be taken during the test period is determined at the beginning of the test. After finishing the test, images are saved in the memory; afterwards, the velocity is measured by the image analysis software ImageJ. Taking the images can be previewed on the touchscreen and the test can be started by clicking run on the script via touchscreen. There is only one script that can control the relay (LED), the servo motor and the camera. With the one-click, the test can be done.

The RMS is optimized to maximize image resolution and automate the system (Figure S1). It was observed that the most ideal choice in the system in terms of image quality and distance to the sample was the V2.1 camera (Figure S1c). After the camera selection, it was determined that the ideal light source to be used colorimetrically in the background is a handmade LED lightbox (Figure S1d).

In this system, 2 different cameras (HQ and V2.1) and 2 different light sources (factory-made and handmade) were compared to optimise image quality. When comparing the HQ camera with the V2.1 camera, we had to put the HQ camera at a minimum distance of 35 cm from the sample. This made the system larger and more inconvenient. While the maximum sensor resolution value of the HQ camera is 4056*3040 pixels, V2.1 is 3280*2464 pixels. However, there was not much difference between the images, and when the distance between the sample and the camera was evaluated, it was determined that the most suitable camera was V2.1. Additionally, the HQ camera takes fisheye images, and this is a feature we did not want. Although the HQ camera image seems better at first glance due to the light source in the background, this issue has been resolved with a handmade light source. It was observed that the images were taken in high quality and allowed for image analysis with the V2.1. This study clearly showed that the choice of the light source is as important as the choice of camera. Since it does not need a separate power supply, the system becomes a single instrument controlled by a single code. Python software can switch on the LED at the beginning of the experiment and switch off it at the end of the experiment. The handmade light source provided a more homogeneous spread of light and improved image quality (Figure S1c). On the other hand, the white light provided by the factory-made LED was not suitable for colorimetric use.

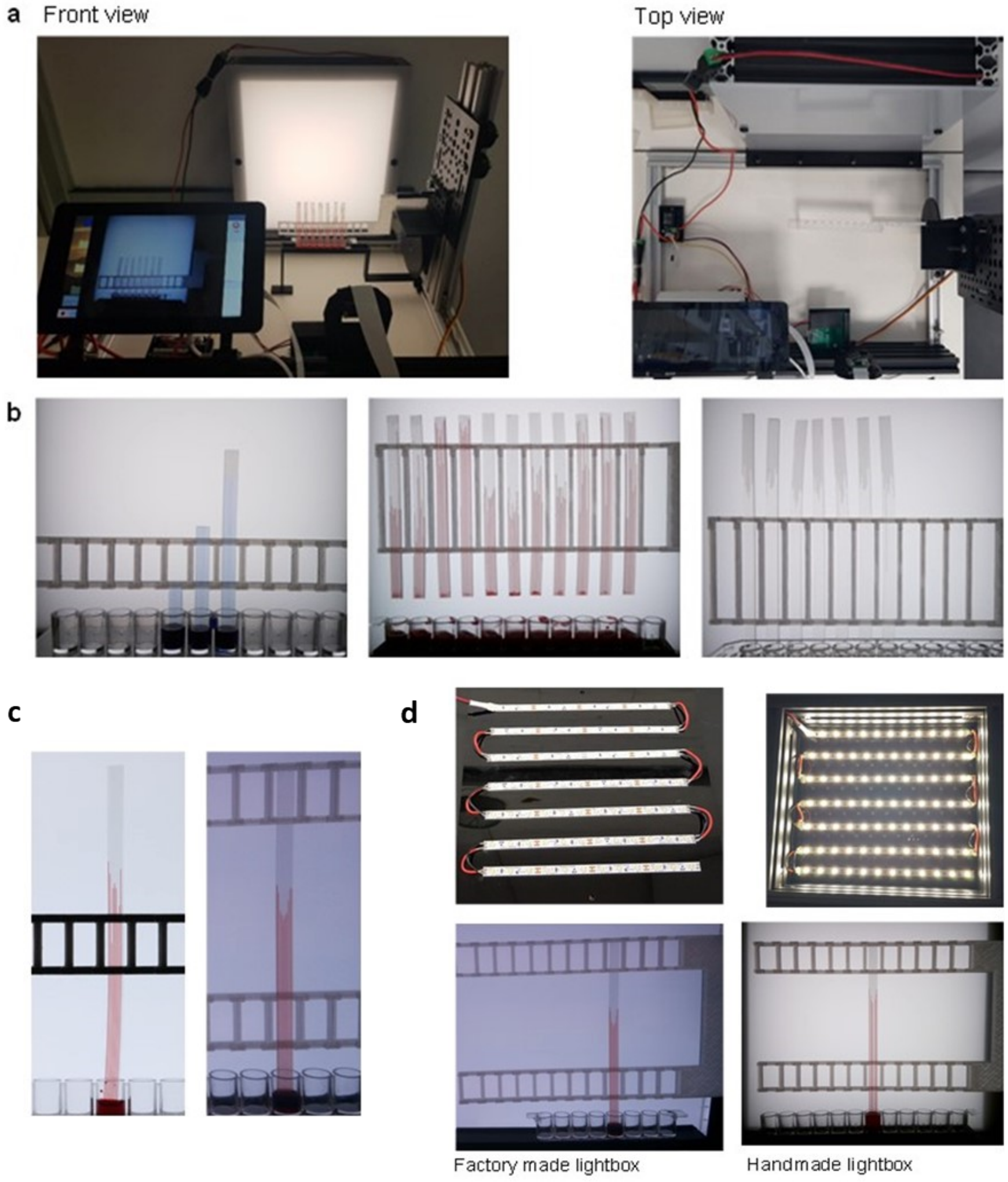

Figure S1. RMS system design and optimisation a) Image of the RMS from front view and top view. The rig has a white lightbox for colorimetric behind the system. 12 MCF strips can be placed between the light box and camera. The dimensions of this rig are 350 x 415 x 288 mm. The camera resolution is 3280x2464. All files needed to build the RMS have been saved on GitLab. b) Different coloured liquids such as resazurin, whole blood and platelet-rich plasma were captured with Raspberry Pi camera V2.1. c) The first image was taken with the HQ camera. The distance between the camera and the sample was 35 cm. The next image was taken with the V2.1 camera. The distance of the test strips from the camera was 17 cm. d) Demonstration of building an LED lightbox and comparing images taken using a factory-made and a handmade lightbox.

S2 Preparation of MCF test strips

Hydrophilic coating protocol

The inner surface of MCF was coated with poly (vinyl alcohol) (PVOH) (Sigma-Aldrich, UK) to gain hydrophilic property. This involved cutting a 1 m long MCF with a sharp blade and connecting it to a KNF Laboport mini vacuum pump (Sigma-Aldrich, UK), and filling it with 10 gL-1 PVOH in distilled water. To prevent evaporation and leakage, 2 ends of the coated MCF were sealed with parafilm and it was incubated for 2 hours at room temperature (RT). After incubation, the PVOH solution was removed using the vacuum pump, then capillaries washed with 0.05% v/v Tween 20 (Sigma-Aldrich,

UK) solution in distilled water. The MCF was left attached to the vacuum pump for 20 minutes to dry.

Reagent loading protocol

1m PVOH coated MCF were cut into 6 - 10cm lengths. Reagent solutions (obtained by 4 or 5 times 3.16-fold dilutions in deionized water with an initial dilution concentration of 0.01M for adenosine 5`diphosphate (ADP) (Sigma-Aldrich, UK) and 500U/mL for Thrombin) at different concentrations were filled into the PVOH coated MCF via vacuum pump. The reagent-filled strips were sandwiched between steel plates and placed in a -80°C freezer for one hour, then placed in a Virtis AdVantage Plus Freeze Dryer (SP Industries Inc., Warminster, PA, USA) for an overnight freeze-drying cycle.

S3 Supplemental tables and figures

The pressure balance model with glycerol was validated by performing dH/dt vs 1/h. The Solver can be used to determine unknown parameters in samples with high accuracy. Unknown properties can be estimated in samples simply by tracking the distance of fluid (Figure S2). We noticed that there were divergences from linearity for dH/dt vs 1/h with the larger 270 μm diameter capillary (Figure S4).

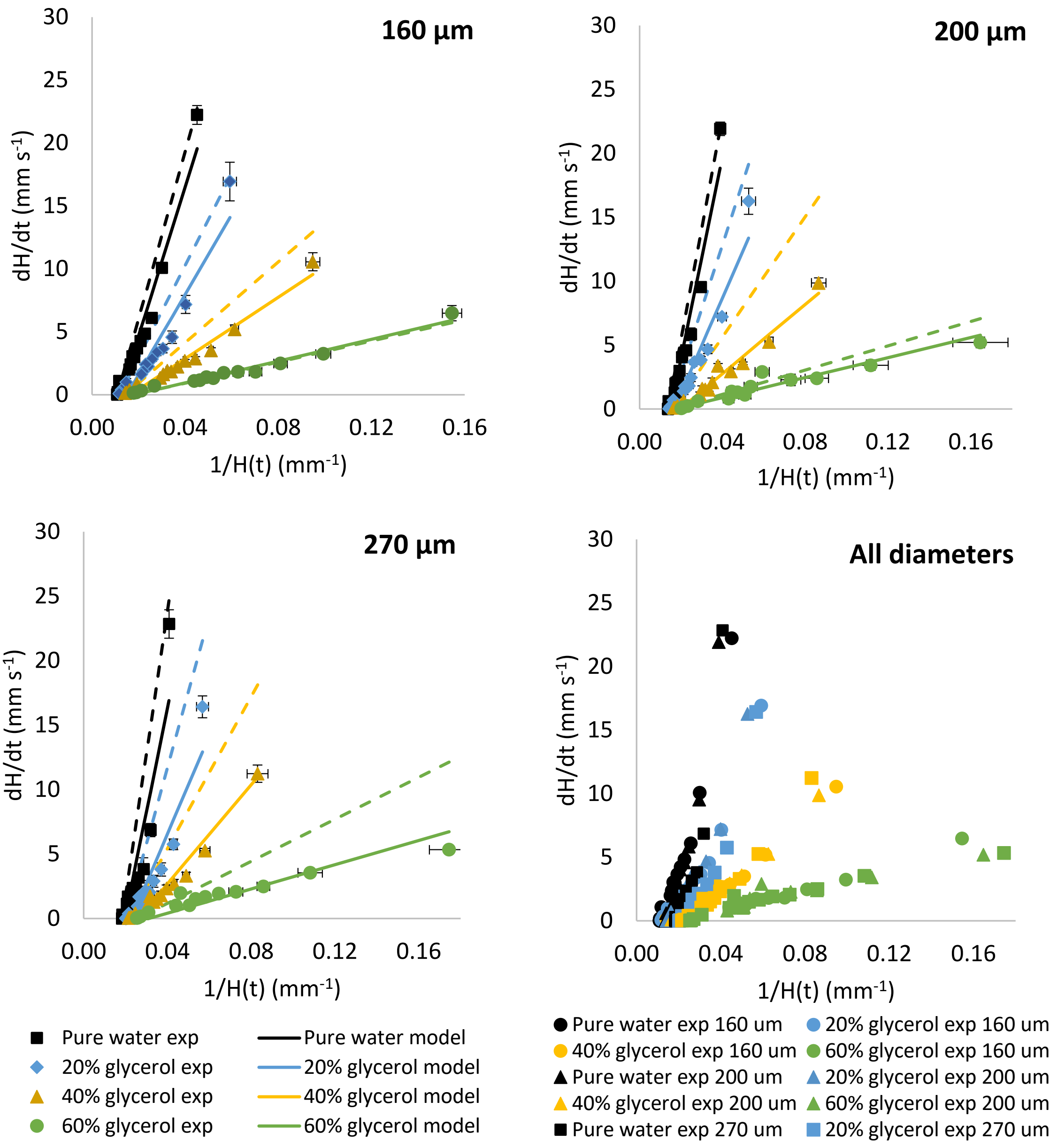

Figure S2. Transient flow of water:glycerol mixtures in the microcapillaries and comparison with dynamic pressure balance model. Comparison of experimental and pressure balance model data for 160, 200 and 270 µm diameter with glycerol water mixtures for validating the model. The solid lines indicate measured values using the pressure balance model and the dashed lines indicate the predicted values.

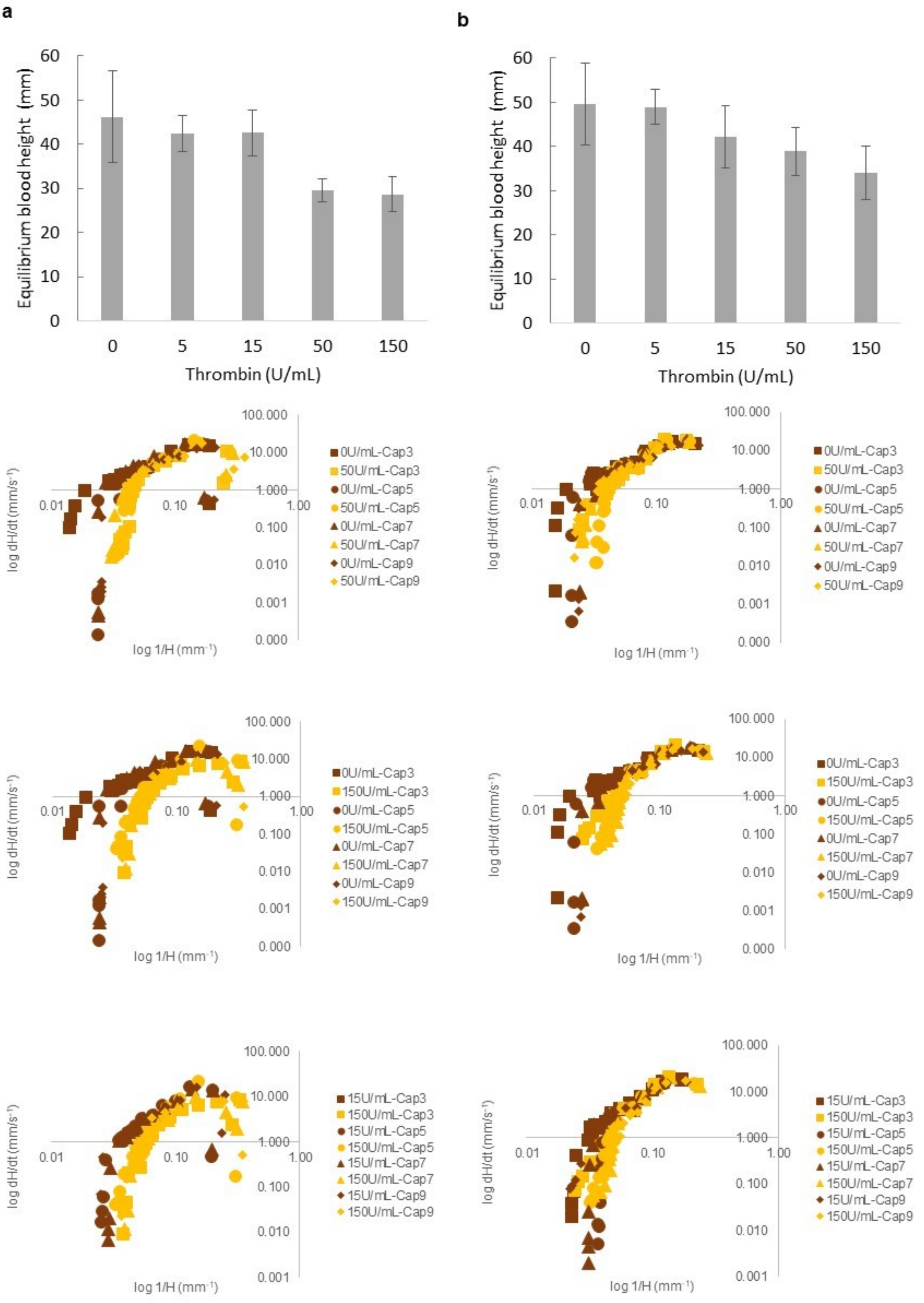

Figure S3. Reproducibility of thrombin-stimulus data within different MCF capillaries for equilibrium height and transient flow using two different donors as shown in (a) and (b).

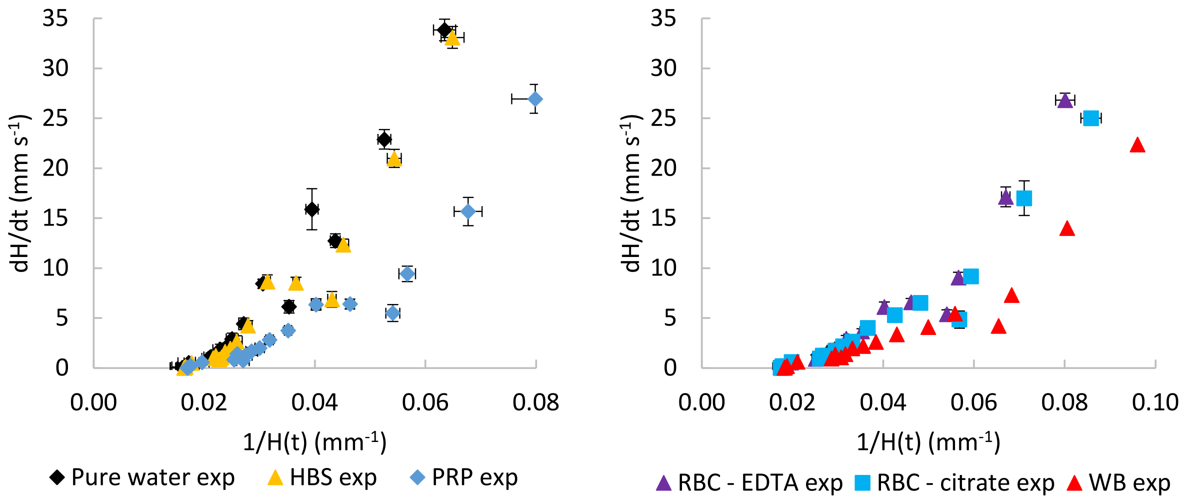

Figure S4. Transient flow of experimental instantaneous superficial fluid velocity dH/dt vs reciprocal H(t) comparing water and buffer with PPP and PRP and comparing WB with washed RBCs for 270 µm inner diameter.

Table S1. Major, minor and hydraulic diameters of individual capillaries of different diameters for PVOH coated MCFs, and maximum equilibrium heights of 4 different glycerol-water mixtures.

					Maximum, equilibrium height, H (cm)			
	**Capillary#**	**Major axis capillary, 2a (µm)**	**Minor axis capillary, 2b (µm)**	**Hydraulic diameter, dh (µm)**	**0% glycerol**	**20% glycerol**	**40% glycerol**	**60% glycerol**
**160µm diameter**	1	154.03	139.66	146.41	10	8.572	7.892	6.414
	2	174.92	156.63	165.14	9.462	8.776	7.116	5.445
	3	158.01	151.42	154.63	9.388	8.317	7.139	5.672
	4	172.36	151.42	161.04	9.048	8.204	7.048	5.49
	5	182.75	172.3	177.33	9.099	7.989	6.963	5.552
	6	170.98	160.54	165.55	8.685	8.238	6.708	5.411
	7	169.2	166.93	168.06	8.929	8.509	7.008	5.547
	8	152.8	142.56	147.46	8.936	8.623	7.02	5.62
	9	173.59	135.75	151.79	9.553	8.634	7.144	5.609
	10	125.33	125.31	125.32	9.547	9.807	6.805	5.649
**200µm diameter**	1	185.74	195.83	190.62	8.322	7.806	6.445	5.71
	2	222.46	189.09	204.09	7.669	7.514	5.803	5.154
	3	215.89	198.88	206.95	7.192	6.928	5.768	4.269
	4	212.42	209.56	210.98	6.732	6.434	5.579	4.93
	5	223.98	213.67	218.67	6.486	6.383	5.705	4.786
	6	199.49	209.86	204.51	6.629	6.492	5.55	4.832
	7	213.55	209.99	211.75	6.847	6.583	6.176	4.884
	8	193.38	190.57	191.96	7.393	7.003	6.48	5.338
	9	195.6	198.74	197.15	8.041	7.657	6.607	5.309
	10	179.66	178.18	178.92	7.737	7.766	6.607	4.672
**270µm diameter**	1	230.54	219.82	225.02	6.102	5.452	5.095	4.37
	2	258.5	198.45	223.56	6.195	5.624	5.13	4.295
	3	254.86	230.15	241.72	5.268	5.054	4.554	3.955
	4	287.33	230.19	254.83	4.812	4.329	4.33	3.627
	5	307.41	282.66	294.39	4.916	4.553	4.243	3.598
	6	318.19	296.56	306.90	5.158	4.652	4.335	3.662
	7	299.26	284.26	291.52	5.227	4.721	4.463	3.812
	8	290.7	266.05	277.69	5.654	4.721	4.393	3.759
	9	258.65	245.67	251.95	6.288	5.706	5.119	4.388

10	269.57	224.78	244.64	5.557	5.338	5.286	4.313

Table S2. Density, surface tension and viscosity for 4 glycerol-water mixtures at T=20°C taken from reference [2].

Mixture	H_2O (wt%)	Glycerol (wt%)	density, ρ (g/cm^3)	surface tension, σ (dyn/cm)	viscosity, μ (cP)
m1	100	0	0.998	73.2	1.04
m2	80	20	1.047	71.7	1.84
m3	60	40	1.1	70	3.63
m4	40	60	1.156	68.5	11.67

Table S3. Density and viscosity properties of water, buffer and blood components from the literature

	Density (g/mL)	Ref	Viscosity range (cP)	Ref
Water	0.998	[2]	1.04	[2]
HBS	0.998	-	1.04	-
PPP	1.025	[3]	1.5-1.72 (25C)	[4]
PRP	1.025	[3]	1.5-1.72 (25C)	[4]
RBC - EDTA	1.030	[3]	2 - 6	[5]
RBC - Citrate	1.030	[3]	2 - 6	[5]
WB	1.055	[3]	2 - 6	[5]

Table S4. Predicted theoretical and experimental measured viscosity values of water:glycerol mixtures with percentage differences

	160 um	200 um	270 um
**Pure water**			
Measured	1.05	1.06	1.34
Predicted	1.04	1.04	1.04
% difference	1.23	1.47	22.55
**20% glycerol**			
Measured	1.66	2.12	2.52
Predicted	1.84	1.84	1.84
% difference	9.57	13.34	27.12
**40% glycerol**			
Measured	4.11	5.15	4.63
Predicted	3.63	3.63	3.63

% difference	11.66	29.51	21.60
**60% glycerol**			
Measured	11.25	13.17	16.67
Predicted	11.67	11.67	11.67
% difference	3.64	11.39	29.99

Table S5. Predicted theoretical and experimental measured surface tension and contact angle values of water:glycerol mixtures with percentage differences

**Surface tension (mN/m²)**				**Contact angle (Θ)**		
	**160 µm**	**200 µm**	**270 µm**	**160 µm**	**200 µm**	**270 µm**
**Pure water**						
Measured	71.40	71.82	70.36	63.42	62.63	62.09
Predicted	73.20	73.20	73.20	61.05	60.59	61.31
% difference	2.45	1.88	3.88	3.73	3.25	1.25
**20% glycerol**						
Measured	66.76	67.51	66.94	66.09	62.40	63.69
Predicted	71.70	71.70	71.70	61.44	59.47	62.18
% difference	6.90	5.84	6.64	7.03	4.69	2.38
**40% glycerol**						
Measured	68.36	68.57	67.89	67.87	66.12	66.22
Predicted	70.00	70.00	70.00	64.72	61.93	61.95
% difference	2.34	2.04	3.01	4.65	6.33	6.45
**60% glycerol**						
Measured	68.47	68.50	66.24	68.71	67.25	68.66
Predicted	68.50	68.50	68.50	68.61	65.42	64.66
% difference	0.05	0.00	-3.41	0.14	-2.79	-6.19

Supplementary References

1. Reis, N.M., et al., *Lab on a stick: multi-analyte cellular assays in a microfluidic dipstick.* Lab on a Chip, 2016. **16**(15): p. 2891-2899.
2. Takamura, K., H. Fischer, and N.R. Morrow, *Physical properties of aqueous glycerol solutions.* Journal of Petroleum Science and Engineering, 2012. **98-99**: p. 50-60.
3. Trudnowski, R.J. and R.C. Rico, *Specific Gravity of Blood and Plasma at 4 and 37 °C.* Clinical Chemistry, 1974. **20**(5): p. 615-616.
4. Stuart, J. and M.W. Kenny, *Blood rheology.* Journal of Clinical Pathology, 1980. **33**(5): p. 417.
5. Khnouf, R., et al., *Microfluidics-based device for the measurement of blood viscosity and its modeling based on shear rate, temperature, and heparin concentration.* Biomedical Microdevices, 2019. **21**(4): p. 80.
